# A randomized trial evaluating efficacy of overminus lenses combined with prism in the children with intermittent exotropia

**DOI:** 10.1186/s12886-021-01839-0

**Published:** 2021-02-06

**Authors:** Yuelan Feng, Jingjing Jiang, Xueqing Bai, Hui Li, Ningdong Li

**Affiliations:** 1 Department of Ophthalmology, National Center for Children’s Health, Beijing Children’s Hospital, Capital Medical University, Beijing, China; 2 Department of Ophthalmology, First Hospital Affiliated to Baotou Medical College, Baotou, China

**Keywords:** Intermittent exotropia, Overminus lenses, Prism

## Abstract

**Background:**

To evaluate the efficacy of overminus lenses combined with prism spectacles in children of 3 to 6 years of age with intermittent exotropia (IXT).

**Methods:**

Sixty patients with IXT were randomly assigned to the treatment and observation groups. Each group included 30 IXT children aged 3 to 6 years. The treatment group was prescribed overminus lenses of − 2.50 D incorporated with the 2 PD base-in prisms on each side. Ocular alignment, the status of binocular vision, as well as the refraction changes were carried out and followed at 1, 3, 6, and 12 months. A revised form of the Newcastle Control Score (NCS) was used to evaluate the patients’ ability to control their IXT.

**Results:**

After 12 months**,** the mean refractive error was 1.42 ± 1.25 D, and 1.43 ± 1.12 D for the observation and the treatment group, respectively (95% CI: − 0.61 to 0.62)); the mean exotropia control score was 5.72 ± 1.28 and 1.75 ± 1.18 in the observation and the treatment group, respectively (95% CI: − 4.63 to − 3.33); the mean near stereoacuity was 2.16 ± 0.42 log arcsec and 1.91 ± 0.26 log arcsec in the observation and the treatment group, respectively (95% CI: − 0.44 to − 0.06).

**Conclusions:**

In our randomized clinical trial, overminus spectacles with prism significantly improved the control of IXT and stereopsis, by reducing the angle of strabismus in children with IXT. This treatment does not appear to cause myopia, at least in the manner used this series. A further randomized trial is warranted to assess the effect of overminus spectacles with prism after the treatment has been discontinued.

**Trial registration:**

This study adheres to CONSORT 2010 guidelines. Chinese Clinical Trial Registry, ChiCTR1900025243. Registered 17 August 2019.

## Background

Intermittent exotropia (IXT) is a very common form of childhood strabismus, occurring in approximately 0.5 to 1% of the general population [1, 2]. It accounts for approximately 25% of pediatric strabismus in Caucasians and that of 44.9% in Chinese children [3]. Early onset of IXT, within the age of 2 to 5 years, is common, but it may also present shortly after birth [4]. Patients with IXT are characterized by an intermittent outward deviation of one or both eyes when fixating on a target at a distance or when fatigued. Surgery is considered to be an effective method for the correction of exotropia and restoration of binocular vision. However, there are some arguments regarding the optimal surgical timing for children. Overminus lenses, as an alternative non-surgical intervention treatment, are often used for children. Studies suggest that overminus therapy stimulates accommodative convergence and helps reduce the angle of exodeviation [5, 6]. Another hypothesis is that the fusional convergence is exerted to control the exodeviation, inducing convergence accommodation and distance blur, and that overminus lenses may allow clear distance vision, facilitating fusion [7]. Prism has also been used to treat IXT as it maintains fusion, alleviates symptoms, and reinforces binocular functions, both alone and in conjunction with orthoptic exercises [8, 9]. Previous studies of IXT conservative therapy were mainly focused on overminus lenses, it appear to be well controlled in the regular refractive correction. Nevertheless, these studies have been limited to retrospective case series without comparison groups and few studies reported the outcomes of overminus lenses combined with prism. Therefore, in this study, we evaluated the efficacy of overminus lenses combined with prism for children aged 3 to 6 years.

## Methods

Sixty patients with IXT were recruited for this study. The inclusion criteria were as follows: (1) Exodeviation angle was more than 15 prism diopters (PD) at a distance measured by prism and alternate cover test (PACT); (2) The difference was within 10 PD between the near and distance deviations; (3) The spherical equivalent (SE) ranged from + 0.50 to + 5.00 D; (4) The Newcastle Control Score (NCS) was ≥3. Patients with neurological disorders who could not cooperate well for ocular examinations, oblique muscle overaction, disassociated vertical deviation or vertical deviations > 5 PD, anisometropia > 2 D, and follow-up time < 12 months, were excluded.

Participants were randomly assigned (using a permuted block design stratifified by exotropia control (0–3, 4–6, 7–9) with equal probability to treatment group or observation.

The treatment group was prescribed with overminus lenses of − 2.50 D incorporated with base-in prisms of 2 PDs on each side after cycloplegic refraction. The observation group was supposed to wear spectacles if refractive error met any of the following criteria: SE anisometropia ≥1.00 D; astigmatism ≥1.00 D in either eye; and SE hyperopia ≥ + 1.00 D [10]. The others who did not need refractive correction were prescribed plano lens spectacles that were to be worn at each follow-up visit but were not to be worn in the interim.

Ocular alignment, status of the binocular vision, and refractive changes were evaluated regularly for each patient at 1 month, 3 months, 6 months, and 12 months. Distance visual acuity (VA) was measured using the Amblyopia Treatment Study HOTV testing protocol and near VA testing using the Amblyopia Treatment Study 4 test (Precision Vision, La Salle, IL). The NCS incorporates subjective (home control) and objective (clinic control at near and in the distance) criteria (Table 1). The home control section asks the parent/guardian to rate the frequency with which the strabismus is noticed to be present, while the clinic components rate the ease with which binocular single vision is regained after a cover test. The exodeviation angle was measured with PACT at both 6 m and 33 cm using an accommodative target. Stereoacuity was assessed using the Randot Preschool Stereoacuity test (Stereo Optical Co., Inc., Chicago, IL) at 40 cm. All patients were assessed by the same study-certifified masked examiner (a pediatric ophthalmologist) at primary and 1-, 3-, 6-, and 12-month outcomes in all cases.

**Table 1:** **The Revised Newcastle Control Score**

Control of exodeviation will be measured at Home and the Clinic using the revised Newcastle Control Score (NCS)	
Distance (6 m): fixing on an accommodative target	
Near (1/3 m): fixing on Lang near-viewing stick or a similar accommodative target	
The scale below applies to both distance and near separately.	
**Home control**	**Score**
XT or monocular eye closure seen never	0
< 50% of time fixing at distance	1
> 50% of time fixing at distance	2
> 50% of time fixing at distance + seen at near	3
**Clinical control**	
**Near**	
Immediate realignment after dissociation	0
Realignment with aid of blinking or re-fixation	1
Remains manifest after dissociation or prolonged fixation	2
Manifest spontaneously	3
**Distance**	
Immediate realignment after dissociation	0
Realignment with aid of blinking or re-fixation	1
Remains manifest after dissociation or prolonged fixation	2
Manifest spontaneously	3
Total NCS = home + near+ distance.	

### Data analysis

The sample size was 55 participants using a 1-sided test with alpha = 0.05, assuming 10% loss to follow up. A priori the study had 80% power to detect a treatment group difference in mean 12-month near stereoacuity, assuming a true difference of − 0.25 points or larger (Overminus and prism minus observation) with a standard deviation of 0.50 (based on prior studies). A 1-sided hypothesis test using alpha = 0.05 was used to determine whether the overminus and prism group had better 12-month mean near stereoacuity than the observation group in this pilot study. Outcome variables were tested for departures from a normal distribution with the Shapiro-Wilk test. If significant departures were found, appropriate nonparametric tests were also used to assess for a treatment effect. Statistical analysis was performed to compare the differences between the treatment and observation groups in terms of refractive changes, control of IXT, exodeviation, and stereoacuity (converted from seconds of arc to log arcsec values). The comparison of the treatment and observation groups was performed using an analysis of covariance (ANCOVA) model that was adjusted for baseline control, baseline refractive error, baseline stereoacuity, baseline distance, and near PACT. The parameters before and after the treatment were compared using parametric (paired *t*-test) analysis. All statistical analyses were performed using SPSS 20.0 (SPSS Inc., Chicago, IL, USA). A *p* value < 0.05 was accepted as significant.

## Ressults

### Baseline characteristics

Sixty Asian patients with IXT were recruited for this study during 2019–2020, 30 of which received the overminus lenses combined with prism treatment and 30 were under observation. The average patient age was 4.75 ± 1.03 years; 28 (46.7%) were female, and 32 (53.3%) were male. The baseline clinical characteristics are shown in Table 2.

**Table 2:** Baseline demographics and clinical characteristics

	Overminus and prism *n* = 30		Observation *n* = 30	
	*N*	%	*N*	%
**Sex**				
Female	13	43.3	15	50.0
Male	17	56.7	15	50.0
**Age**				
3 to < 4	3	10.0	4	13.3
4 to < 5	8	26.7	10	33.3
5 to < 6	9	30.0	9	30.0
6 to < 7	10	30.0	7	23.3
Mean (SD)	4.87 ± 1.01		4.63 ± 1.00	
Range	3 to 6		3 to 6	
**Refractive error (SE, D)**				
+ 4.00 to <+ 5.00	1	3.3	2	6.7
+ 3.00 to <+ 4.00	2	6.7	4	13.3
+ 2.00 to <+ 3.00	3	10.0	5	16.7
+ 1.00 to <+ 2.00	9	30.0	6	20.0
+ 0.5 to <+ 1.00	15	50.0	13	43.3
Mean (SD)	1.59 ± 1.05		1.64 ± 1.29	
Range	+ 0.5 to + 4.5		+ 0.5 to + 4.25	
**Exotropia control**				
0 to 3	2	6.7	5	16.7
4 to 6	20	66.7	19	63.3
7 to 9	8	26.7	6	20.0
Mean (SD)	5.37 ± 1.65		5.60 ± 1.50	
Range	2 to 8		2 to 8	
**Exodeviation by PACT at distance (PD)**				
1 to 9	0	0.0	0	0.0
10 to 14	0	0.0	0	0.0
16 to 18	6	20.0	6	20.0
20 to 25	10	33.3	8	26.7
30 to 35	10	33.3	13	43.3
40 to 45	4	13.3	3	10.0
Mean (SD)	27.80 ± 7.48		27.46 ± 7.60	
Range	16 to 40		16 to 40	
**Exodeviation by PACT at near (PD)**				
1 to 9	0	0.0	0	0.0
10 to 14	1	3.3	1	3.3
16 to 18	7	23.3	7	23.3
20 to 25	10	33.3	12	40.0
30 to 35	9	30.0	9	30.0
40 to 45	3	10.0	1	3.3
Mean (SD)	25.13 ± 6.84		26.46 ± 8.08	
Range	14 to 40		14 to 40	
**Preschool Randot near stereoacuity, arcsec (log arcsec)**				
40″ (1.6 log arcsec)	3	10.0	4	13.3
60 ~ 80″ (1.78 log arcsec)	7	23.3	9	30.0
100 ~ 140″ (2.0 log arcsec)	6	20.0	5	16.7
200″ (2.3 log arcsec)	5	16.7	4	13.3
400″ (2.6 log arcsec)	5	16.7	3	10.0
800″ (2.9 log arcsec)	3	10.0	4	13.3
Nil (3.1 log arcsec)	1	3.3	1	3.3
Mean (SD) (log arcsec)	2.14 ± 0.46		2.19 ± 0.44	
Range (log arcsec)	1.6 to 3.1		1.6 to 3.1	

### Visit completion

The 1-month and 3-month visits were completed by all 60 patients, and 29 patients (96.7%) and 30 patients (100%) in the treatment and observation groups, respectively, completed the 6-month visit. Twenty-eight of 30 (93.3%) and 30 (96.7%) patients in the treatment and observation groups, respectively, completed the 12-month visit.

### Analysis of the baseline and final follow-up measurements between the treatment and observation groups

The mean refractive error, exotropia control, deviation magnitude by the PACT, and near stereoacuity for the participants who completed the 1-, 3-, 6-, and 12-month visits are listed in Table 3. The mean refractive error was 1.42 ± 1.25 D in the observation group and 1.43 ± 1.12 D in the treatment group at 12 months (95% CI: −0.61 to 0.62). The mean exotropia control was 5.72 ± 1.28 in the observation group and 1.75 ± 1.18 in the treatment group (95% CI: −4.63 to −3.33). 71.4% patients showed an NCS of 3 or less in the treatment group compared with 6.89% in the observation group. There was a signficant reduction in the angle of deviation in the treatment group, especially at near. For observation and treatment groups respectively, 12-month outcomes showed that mean near PACT measurements were 26.72 ± 7.41PD vs. 16.25 ± 7.03PD (95% CI: −14.31 to 6.64). The improvement in near stereoacuity of the final evaluation was 2.16 ± 0.42 log arcsec in the observation group and 1.91 ± 0.26 log arcsec in the treatment group (95% CI: − 0.44 to − 0.06) (Table 4).

**Table 3:** The mean orthoptic measurements at each follow-up points

	Overminus and prism	Observation	*p* value
**Refractive error (SE, D)**			
pretreatment	1.59 ± 1.05	1.64 ± 1.29	0.254
1 month	1.59 ± 1.05^#^	1.64 ± 1.29^##^	0.254
3 months	1.34 ± 1.08^#^	1.54 ± 1.29^##^	0.342
6 months	1.34 ± 1.08^#^	1.48 ± 1.25^##^	0.271
12 months	1.43 ± 1.12^#^	1.42 ± 1.25^##^	0.353
**Exotropia control**			
pretreatment	5.37 ± 1.65	5.60 ± 1.50	0.569
1 month	3.70 ± 1.26^*^	5.50 ± 1.43^##^	< 0.01
3 months	2.38 ± 0.98^*^	5.67 ± 1.30^##^	< 0.01
6 months	2.17 ± 1.00^*^	5.76 ± 1.28^##^	< 0.01
12 months	1.75 ± 1.18^*^	5.72 ± 1.28^##^	< 0.01
**Exodeviation by PACT at distance (PD)**			
pretreatment	27.80 ± 7.48	27.46 ± 7.60	0.865
1 month	22.80 ± 7.50^*^	27.80 ± 8.14^##^	0.017
3 months	22.72 ± 7.62^*^	28.03 ± 8.16^##^	0.012
6 months	22.55 ± 7.62^*^	28.83 ± 8.23^##^	0.004
12 months	22.46 ± 7.61^*^	27.48 ± 7.24^##^	0.014
**Exodeviation by PACT at near (PD)**			
pretreatment	25.13 ± 6.84	26.46 ± 8.08	0.493
1 month	16.93 ± 7.09^*^	26.76 ± 8.19^##^	< 0.01
3 months	16.37 ± 6.11^*^	27.00 ± 8.20^##^	< 0.01
6 months	16.13 ± 5.87^*^	26.66 ± 7.63^##^	< 0.01
12 months	16.25 ± 7.03^*^	26.72 ± 7.41^##^	< 0.01
**Preschool Randot near stereoacuity, arcsec (log arcsec)**			
pretreatment	2.14 ± 0.46	2.19 ± 0.44	0.717
1 month	1.97 ± 0.33^*^	2.19 ± 0.44^##^	0.03
3 months	1.96 ± 0.29^*^	2.19 ± 0.44^##^	0.021
6 months	1.95 ± 0.28^*^	2.17 ± 0.42^##^	0.017
12 months	1 .91 ± 0.26^*^	2.16 ± 0.42^##^	0.01

*Compared with pretreatment in treatment group, *p*<0.05; ^#^Compared with pretreatment in treatment group, *p*>0.05; ^##^Compared with pretreatment in observation groups, *p*>0.05

**Table 4:** Orthoptic measurements between treatment group and observation group after 12 months

	Overminus and prism *n* = 28		Observation *n* = 29	
	*n*	%	*n*	%
**Refractive error (SE, D)**				
+ 4.00 to <+ 5.00	1	3.6	2	6.9
+ 3.00 to <+ 4.00	2	7.1	3	10.3
+ 2.00 to <+ 3.00	4	14.3	7	24.1
+ 1.00 to <+ 2.00	10	35.7	7	24.1
0 to <+ 1.00	10	35.7	10	34.5
-1.00 to<0	1	3.6	0	0.0
Mean (SD)	1.43 ± 1.12		1.42 ± 1.25	
Range	−0.25 to + 4.5		+ 0.25 to + 4.25	
Difference in means (95% CI)	0.01 (−0.61 to 0.62)			
Change between baseline and 12 months	*p* = 0.278		*p =* 0.378	
**Exotropia control**				
0 to 3	20	71.4	2	6.9
4 to 6	6	21.4	19	65.5
7 to 9	2	7.1	8	27.6
Mean (SD)	1.75 ± 1.18		5.72 ± 1.28	
Range	3 to 8		3 to 8	
Difference in means (95% CI)	−3.97 (−4.63 to −3.33)			
Change between baseline and 12 months	*p<*0.001		*p =* 0.546	
**Exodeviation by PACT at distance**				
No exodeviation (orthophoria)	0	0.0	0	0.0
1 to 9	0	0.0	0	0.0
10 to 14	6	21.4	4	13.8
16 to 18	3	10.7	11	37.9
20 to 25	11	39.3	11	37.9
30 to 35	8	28.6	3	10.3
40 to 45	0	0.0	8	28.6
Mean (SD)	22.46 ± 7.61		27.48 ± 7.24	
Range	12 to 35		18 to 45	
Difference in means (95% CI)	−5.02 (−8.96 to −1.08)			
Change between baseline and 12 months	*p*<0.001		*p =* 0.177	
**Exodeviation by PACT at near**				
No exodeviation (orthophoria)	0	0.0	0	0.0
1 to 9	4	14.3	0	0.0
10 to 14	6	21.4	6	20.7
16 to 18	8	28.6	11	37.9
20 to 25	8	28.6	10	34.5
30 to 35	2	7.1	2	6.9
40 to 45	0	0.0	0	0.0
Mean (SD)	16.25 ± 7.03		26.72 ± 7.41	
Range	8 to 30		16 to 40	
Difference in means (95% CI)	−10.47 (−14.31 to −6.64)			
Change between baseline and 12 months	*p*<0.001		*p =* 0.127	
**Preschool Randot near stereoacuity, arcsec (log arcsec)**				
40″ (1.6 log arcsec)	7	25	3	10.3
60″ (1.78 log arcsec)	8	28.6	7	24.1
100″ (2.0 log arcsec)	8	28.6	6	20.7
200″ (2.3 log arcsec)	4	14.3	6	20.7
400″ (2.6 log arcsec)	1	3.6	3	10.3
800″ (2.9 log arcsec)	0	0.0	4	13.8
Nil (3.1 log arcsec)	0	0.0	0	0.0
Mean (SD) (log arcsec)	1 .91 ± 0.26		2.16 ± 0.42	
Range (log arcsec)	1.6 to 2.6		1.6 to 2.9	
Difference in means (95% CI)	−0.25 (−0.44 to −0.06)			
Change between baseline and 12 months	*p*<0.001		*p =* 0.265	

## Discussion

The management of IXT is unlike other types of childhood onset strabismus as the timing and method of intervention are controversial. Many studies have advocated for a delayed management because it may help in avoiding a higher overcorrection rate and poor sensory outcomes caused by early surgery while also allowing for more accurate measurements and better results [11, 12]. It has been suggested that surgical outcomes of IXT are optimized if combined with non-surgical treatment, which may be more effective in preschool children. Hence, conservative options have been deemed useful in this “delay” period.

The use of prisms has been primarily focused on pre- or postsurgical applications to facilitate binocular sensory fusion, but there are few studies on its management of IXT. In our study, we prescribed 2 D base-in prism spectacles OU instead of base-out prism because the base-out prism would be used as an “exercise” for fusional convergence, which might seem useful for the treatment of exotropia. However, the base-out prism, in effect, increases the size of the deviation and thus, makes fusion more difficult. It may not only be useful for dedicated exercise sessions but also result in frequent manifestation of the deviation. The prescribed base-in prisms (relieving or demand-reducing prisms) could neutralize a certain amount of exodeviation, reducing the “demand” on fusional vergence and making its control easier. Additionally, reduced deviation allows sensory fusion even while the visual axes remain diverted (neutralizing prisms). Both of these approaches are intended to partially compensate for an exodeviation in an effort to attain a continuous binocular sensory fusion.

Overminus lenses were often used alone in previous studies [13–16]; their prescription ranged from 0.50 D to 5.00 D considering the children’s adaptability and the risk of myopia caused by overcorrection [15, 17, 18]. In our clinical trial, we chose −2.50 D incorporated with the 2 PD base-in prisms as the initial power of overminus lenses, as it allowed the children to adapt to the lenses to maintain a constant accommodative demand and clear visual quality. We calculated that 4 D of the base-in prism could provide as many accommodative convergence benefits as would 1 D of additional overminus, assuming a normal accommodative convergence to accommodation ratio. Thus, − 2.5 D of overminus with 4 D of base-in prism could achieve the same effect as that of 3.5 D of overminus; this is theoretically less likely to cause asthenopia, especially at near work.

It has been reported that the accommodative efforts of overminus lenses may induce myopia or enhance myopic progression [19]. Some studies have suggested that myopic patients overminused for exotropia became more myopic, and demonstrated that the shift toward myopia is greater in initially myopic than in hyperopic children [20–22]. Considering these hypotheses, we chose children with hyperopia as subjects. After 12 months of follow-up, 92% of the patients had no change in their refractive error, 5% of the children had a small decrease, which was less than + 0.5 D. The refractive error was not significantly different between the treatment and observation groups, and before and after treatment in the treatment group. In fact, previous studies on children with intermittent exotropia have suggested that overminus lenses treatment does not cause myopic progression [5, 14]. Our research further provided evidence that overminus lenses combined with prism do not cause myopia, at least in the manner used in this series at 12 months follow-up.

In previous studies, overminus treatment significantly improved exotropic control [13–16]. Watts et al. reported that patients with a baseline NCS of ≤4 showed a slightly better exotropic control (75%) compared to patients with a baseline NCS of ≥5 (62.5%) [16]. In our study, we found that 28 of 30 (93.3%) and 20 of 28 (75.4%) patients with a baseline NCS ≥4, showed an NCS ≤ 3 or less after 12-month follow-up in the treatment group, which suggests a significant improvement. Therefore, we consider that even in patients with high NCS, overminus and prism lenses may be an effective method for improving the control of exotropia.

Most studies on overminus focused on the changes in refractive error, magnitude of the exodeviation, and control score, but there were few assessments of binocular visual function. However, it is intuitive to consider that better stereopsis must be associated with good control of IXT and a smaller angle of deviation. In our study, we evaluated exodeviation and stereoacuity in the treatment and observation groups as well as self-control in the treatment group. The decrease in exodeviation is significant, which may be attributed to the overminus and neutral prism. Caltrider and Jampolsky [23] reported that 26% of 35 subjects (aged 2 to 13 years) had a decrease of at least 15PD of exodeviation when wearing overminus lenses (2.00. 4.00 D). In our study, the deviation angle was 25.13 ± 6.84PD and 16.25 ± 7.03PD before and after treatment, respectively (*p* < 0.001). The near stereoacuity in the treatment group was 1.91 ± 0.26 log arcsec versus 2.16 ± 0.42 log arcsec in the observation group at 12 months (95% CI: − 0.25 (− 0.44 to − 0.06)). The improvement of near stereoacuity was also observed in the treatment group (*p* < 0.001). Thus, we think that an overminus-lens combined with prism can reduce the angle of deviation and improve the quality of fusion, and increase binocular visual function.

There are a number of limitations to our study. We only chose children with hyperopia as subjects and did not include the effects of overminus lens and prism in myopic patients. A further randomized clinical trial needs to be conducted. We used the Newcastle Control Score to evaluate exotropia control, which incorporates parental observations of the deviation, which may have an influence on management decisions. This is a clinical study involving patients aged 3 years; therefore, there are limitations in patients’ response to cooperative testing.

## Conclusions

The overminus lenses combined with prism may be a safe initial therapy in children aged 3 to 6 years with IXT. The results show better control of IXT and improved stereoacuity in the treatment cohort, relative to the observational cohort. A further randomized trial is warranted to assess the effect of overminus spectacles with prism after the treatment has been discontinued.

## Data Availability

The datasets used and analysed during the current study are available from the corresponding author on reasonable request.

## Abbreviations

IXT: Intermittent exotropia
NCS: Newcastle Control Score
PD: Prism diopters
SE: Spherical equivalent
PACT: Prism and alternate cover test
ANCOVA: Analysis of covariance
